# Aberrant Topological Patterns of Structural Cortical Networks in Psychogenic Erectile Dysfunction

**DOI:** 10.3389/fnhum.2015.00675

**Published:** 2015-12-18

**Authors:** Lu Zhao, Min Guan, Xiaobo Zhu, Sherif Karama, Budhachandra Khundrakpam, Meiyun Wang, Minghao Dong, Wei Qin, Jie Tian, Alan C. Evans, Dapeng Shi

**Affiliations:** ^1^McConnell Brain Imaging Centre, Montreal Neurological Institute, McGill UniversityMontreal, QC, Canada; ^2^Department of Radiology, Henan Provincial People's HospitalZhengzhou, China; ^3^Department of Urology, Henan Provincial People's HospitalZhengzhou, China; ^4^Douglas Mental Health University Institute, McGill UniversityMontreal, QC, Canada; ^5^School of Life Science and Technology, Xi'Dian UniversityXi'an, China; ^6^Institute of Automation, Chinese Academy of SciencesBeijing, China

**Keywords:** male sexual arousal, magnetic resonance imaging, network topology, graph theory, psychogenic erectile dysfunction

## Abstract

Male sexual arousal (SA) has been known as a multidimensional experience involving closely interrelated and coordinated neurobehavioral components that rely on widespread brain regions. Recent functional neuroimaging studies have shown relation between abnormal/altered dynamics in these circuits and male sexual dysfunction. However, alterations in the topological^1^ organization of structural brain networks in male sexual dysfunction are still unclear. Here, we used graph theory^2^ to investigate the topological properties of large-scale structural brain networks, which were constructed using inter-regional correlations of cortical thickness between 78 cortical regions in 40 patients with psychogenic erectile dysfunction (pED) and 39 normal controls. Compared with normal controls, pED patients exhibited a less optimal global topological organization with reduced global and increased local efficiencies. Our results suggest disrupted neural integration among distant brain regions in pED patients, consistent with previous reports of impaired white matter structure and abnormal functional integrity in pED. Additionally, disrupted global network topology in pED was observed to be primarily relevant to altered subnetwork and nodal properties within the networks mediating the cognitive, motivational and inhibitory processes of male SA, possibly indicating disrupted integration of these networks in the whole brain networks and might account for pED patients' abnormal cognitive, motivational and inhibitory processes for male SA. In total, our findings provide evidence for disrupted integrity in large-scale brain networks underlying the neurobehavioral processes of male SA in pED and provide new insights into the understanding of the pathophysiological mechanisms of pED.

## Introduction

Erectile dysfunction (ED), also known as impotence, is characterized by the persistent inability to develop or maintain penile erection sufficient for successful sexual performance (NIH, 1993). As a common male sexual disorder with high prevalence and incidence (Feldman et al., 1994; Aytaç et al., 1999), ED can have profound adverse effects on the quality of life and the psychological well-being of both sufferers and their partners (Guest and Das Gupta, 2002; Latini et al., 2006). The well-known Massachusetts Male Aging Study reported that more than 50% of men from 40 to 70 years old were affected by ED (Feldman et al., 1994). Psychogenic erectile dysfunction (pED) is the classification of ED where erection fails predominantly or exclusively due to psychological factors (Lizza and Rosen, 1999; Rosen, 2001), such as depressed mood, loss of self-esteem, anxiety, and other psychosocial stresses (Caspari et al., 1999; Aydin et al., 2001; Karadeniz et al., 2004), rather than physical impairments. Epidemiologic studies have found exclusively psychogenic etiology in about 40% of men with ED (Feldman et al., 1994; Caspari et al., 1999; Aydin et al., 2001); this rate reaches up to about 70% in the affected men under 40 years (Mellinger and Weiss, 1992; Caskurlu et al., 2004; Karadeniz et al., 2004).

Brain has been known to play a central role in the control of male sexual function (McKenna, 1999; Argiolas and Melis, 2005). Functional neuroimaging studies assessed the brain responses of healthy subjects to sexually relevant stimuli, mostly visual, and consistently reported distributed cortical and subcortical activations and deactivations involved in normal male sexual arousal (SA) (Kühn and Gallinat, 2011; Stoléru et al., 2012; Sescousse et al., 2013; Poeppl et al., 2014). These multiple regional brain responses in different stages of male SA have been conceived to be organized into a four-component neurobehavioral model of male SA comprising of a cognitive, motivational, emotional, and autonomic and neuroendocrine component (Stoléru et al., 1999, 2012; Redouté et al., 2000). These components are believed to be closely interrelated and coordinated, and appear to be controlled by inhibitory processes. The cognitive component comprises an appraisal process through which stimuli are evaluated as sexual incentives, increased attention to stimuli evaluated as sexual, and motor imagery related to sexual behavior. Functional activation of the orbitofrontal cortex (OFC), the inferior temporal cortex (ITC), the superior parietal lobules (SPL), and regions belonging to the motor imagery network [inferior parietal lobules (IPL), ventral premotor area (vPM), supplementary motor areas (SMA), and cerebellum] are conceived as the neural correlates of the cognitive component. The cognitive appraisal component is postulated as the first step in the whole procedure of male SA development, with later processes depending on it. The emotional component refers to the specific hedonic quality of rising arousal and with the perception of specific bodily changes, such as penile tumescence. Activations of the primary somatosensory cortex that receives inputs from the external genitalia, the left secondary somatosensory cortex, the amygdalas, and the insula are conceived as the neural correlates of the emotional component. The motivational component relates to the processes that direct behavior to a sexual goal, including the perceived urge to express overt sexual behavior. It is suggested that the anterior middle cingulate cortex (MCC), the posterior parietal cortex [Brodmann area 40/supramarginal gyri (SMG)], the hypothalamus, the nucleus accumbens and the substantia nigra participate in the mediation of the motivational component. The autonomic and neuroendocrine component includes various responses, e.g., cardiovascular, respiratory, and genital, leading the subject to a state of physiological readiness for sexual behavior. Activations of the anterior cingulate cortex (ACC), the anterior insula, the putamens and the hypothalamus are thought to mediate the autonomic component. Moreover, it has been revealed that the development of SA in healthy men is always accompanied by deactivations in certain brain regions, such as the medial OFC, the left lateral OFC, parts of the lateral temporal cortex (LTC), the angular cortex (ANG), the posterior cingulate cortex (PCC), and the precuneus (PCUN) (Stoléru et al., 2012; Poeppl et al., 2014). This suggests that these regions may exert continuous inhibitory controls on SA in the absence of sexual stimulation to avoid disadvantages that might ensue from a sexual response (Bancroft et al., 2009); and the development of SA requires release of such inhibition (Stoléru et al., 2003). The brain regions related to the neurobehavioral components and inhibitory control of male SA are summarized in Table 1. For more details about the neurobehavioral model of male SA, see (Stoléru et al., 1999; Redouté et al., 2000; Stoléru et al., 2012).

**Table 1:** **Brain regions related to cognitive, emotional, motivational, autonomic components, and inhibitory control of normal male sexual arousal, which were reported by meta-analyses in Stoléru et al. (2012) and Poeppl et al. (2014)**.

**Cognitive**	**Emotional**	**Motivational**	**Autonomic**	**Inhibitory**
Right lateral OFC, ITC, SPL, IPL, PMv, SMA	SI, SII, pINS, amygdala	PPC, aMCC, claustrum, hypothalamus, SN, striatum	ACC, aINS, putamens, hypothalamus	LTC, medial OFC, dmPFC, PCC, left lateral OFC, ANG

*OFC, orbitofrontal cortex; ITC, inferior temporal cortex; IPL/SPL, inferior/superior parietal lobule; PMv, ventral premotor area; SMA, supplementary motor area; SI/SII, primary/secondary somatosensory cortex; PPC, posterior parietal cortex (Brodmann area 40); aMCC, anterior middle cingulate cortex; SN, substantia nigra; ACC, anterior cingulate cortex; aINS/pINS, anterior/posterior insula; LTC, lateral temporal cortex; dmPFC, dorsal medial prefrontal cortex; PCC, posterior cingulate cortex; ANG, angular gyri*.

Few functional neuroimaging studies have found relation between the decline in male sexual functioning and aberrant brain dynamics in SA (Montorsi et al., 2003; Stoléru et al., 2003; Redouté et al., 2005; Cera et al., 2012b). In a previous study investigating the *in vivo* effect of apomorphine sublingual (an approved medication for ED treatment) on brain activations during visual sexual stimulation using functional magnetic resonance imaging (fMRI) (Montorsi et al., 2003). Montorsi et al. roughly reported extended activations in the cingulate cortex, and the mesial and basal frontal cortex in pED compared with healthy controls. These extended activations were modulated by apomorphine administration which produced a similar dynamic map to that of the healthy controls. Such findings suggest that there might be an involvement of extended inhibitory control on SA development in pED (Stoléru et al., 2003). fMRI findings from a recent study (Cera et al., 2012b) supported this hypothesis. It showed that increased activations in the ventromedial prefrontal cortex (vmPFC), PCC and SPL, regions that have been reported to mediate the inhibitory control of unfolding SA in pED patients compared with healthy subjects. Additionally, decreased activations in the insula, ACC and the hippocampus in pED patients in comparison with healthy subjects during visual sexual stimulation, were observed, which may indicate impairments of the autonomic, cognitive, and motivational processes for SA in pED (Cera et al., 2012b). In addition to the abnormal brain dynamics in male SA, pED may be also associated with aberrant intrinsic central dynamics. A recent study using resting-state fMRI revealed abnormal resting-state activities in multiple brain regions that were conceived to mediate the neurobehaviors and inhibition of male SA, such as the insula, ITC, OFC, MCC, the putamen, the parahippocampal gyrus, LTC, SMA, PCUN, SPL, and the cerebellum (Liu et al., 2015).

As discussed before, male SA is a multidimensional experience comprising closely interrelated and coordinated neurobehavioral components that rely on widespread brain regions. In a recent study (Cera et al., 2014), Cera and colleagues found that pED patients may be associated with disrupted functional connectivities during visual sexual stimuli. The dynamic emergence of coherent physiological activities that span distinct brain regions make up functional networks, and are supported by complex structural networks comprised of neuronal elements (Bullmore and Sporns, 2009). Although, pED has been commonly considered as a functional disorder, the pathophysiology of pED also involves structural brain alterations (Cera et al., 2012a; Zhang et al., 2014; Zhao et al., 2015). For example, a recent study that used structural magnetic resonance imaging (MRI) observed gray matter (GM) atrophy of nucleus accumbens and hypothalamus in pED, which is believed to affect the motivational component of male SA (Cera et al., 2012a). Another study using diffusion-based imaging (DTI) reported microstructural changes in multiple white matter (WM) tracts (Zhang et al., 2014), indicating impairments in neuroanatomical connectivities in pED. In one of our earlier studies (Zhao et al., 2015), we first explored pED-related neuroanatomical abnormalities using a scale space search based brain morphometric analysis (Zhao et al., 2013) in a sample of 40 pED patients and 39 healthy controls. We found that compared with healthy men, pED patients showed significantly decreased cortical thickness (CTh) in multiple cortical regions previously implicated with abnormal dynamics of male SA in pED, such as the medial PFC, OFC, cingulate cortex, ITC, and insula; and CTh reductions in these areas were significantly correlated with male sexual functioning degradation. Moreover, we also investigated structural brain covariance networks in pED. In comparison with healthy controls, pED patients showed decreased inter-regional CTh correlations from the right lateral OFC to the right SMG, and the left ANG, implying disassociation between the cognitive, motivational and inhibitory networks of male SA in pED. In total, the existing neuroimaging evidence suggests that the aberrant brain dynamics related to the pathology of pED, might be associated with disruptions in the architecture of brain networks.

In order to better understand brain networks, network properties should be translated into mathematical metrics. Graph theory, which is a branch of mathematics dealing with the quantitative description and analysis of graphs (networks) represented as sets of nodes and connections, is an ideal tool for such analysis. Graph theoretical analyses on neuroimaging data have demonstrated that the human brain has a small-world organization (Bassett and Bullmore, 2006; Bullmore and Sporns, 2009), i.e., neural networks are optimized for both integrated and localized information processing while minimizing the wiring costs and maximizing the efficiency of information propagation (Latora and Marchiori, 2001; Achard and Bullmore, 2007). Moreover, graph theory-based brain network analysis offers a powerful technique to investigate cognitive and affective dysfunction in diseased brains. Various psychiatric and neurological diseases, e.g., Alzheimer's disease (He et al., 2008), schizophrenia (Zhang et al., 2012), autism (Lewis et al., 2014), and multiple sclerosis (Shu et al., 2011), have been revealed as disorders of brain network organization, especially with aberrant small-world characteristics.

In this work, we aimed to extend our work in Zhao et al. (2015) to explore alterations in the topological patterns of large-scale structural brain networks in pED by implementing graph theoretical analysis on the same data sample. We constructed the structural brain networks by using inter-regional correlation of cortical thickness (CTh) to define the connectivity between each pair of brain regions, known as structural covariance networks (SCN) (Evans, 2013). Numerous existing works have demonstrated that inter-regional covariances in CTh, i.e., CTh of one brain region changes in a statistically correlated fashion with CTh changes in other regions, are partly associated with known neuroanatomical pathways in the human brain (Lerch et al., 2006; He et al., 2007; Gong et al., 2012). Nevertheless, CTh correlations also contain unique information. Since CTh correlations may arise from genetic, maturational and functional interaction effects (Evans, 2013), the examination of SCNs of CTh can possibly contribute to the understanding of functional connectivities (Clos et al., 2014). Considering the existing findings of functional and structural deficits in widespread brain regions mediating controls and processes of male SA and abnormal brain connectivities in pED, here, we hypothesized that the pathophysiology of pED might be correlated with altered brain network topology, i.e., changes in graph theoretical metrics of brain SCNs.

## Materials and methods

### Subjects

This study recruited 40 right-handed male patients diagnosed as pED (mean age = 28.5 ± 6.4 years) and 39 right-handed heterosexual healthy men (mean age = 30.0 ± 3.4 years) at the Henan Provincial People's Hospital. All patients had experienced erectile dysfunction for more than 6 months and were in a stable heterosexual relationship for at least 1 year. All normal controls were free from any urosexual symptoms or signs.

The diagnosis of pED (generalized type) was conducted following current guidelines (Wespes et al., 2006): absence of organic (vasculogenic, neurogenic, hormonal, anatomical, and drug-induced) erectile dysfunction. All patients received physical examinations including penile duplex Doppler ultrasonography for vascular impairment, RigiScan test for Nocturnal Penile Tumescence, electrocardiogram exam. They also received laboratory tests including thyroid-stimulating hormone level, prostate-specific antigen and serum sexual hormone status, specifically for genitourinary, endocrine, vascular, and neurological systems. Detailed records of medical, sexual and relevant drug history and surgical disorders were also acquired from each patient. Twenty three patients (out of Sixty three) were excluded from this study because some of them had experience of ED less than 6 months, or were diagnosed having premature ejaculation, or had hormonal defects, vascular or genital impairments, or had been using medication that might affect sexual function or that was designed to enhance sexual performance during the previous 14 days before the scanning. The same clinical examinations were performed on all the healthy subjects.

The Mini International Neuropsychiatric Interview (M.I.N.I) (Sheehan et al., 1998) and the Brief Psychiatric Rating Scale (BPRS) (Overall and Gorham, 1962) were implemented to all the participants to examine if any of them have one or more psychiatric disorders. None of the participants met the criteria of other DSM-IV disorders than pED or showed prominent psychiatric symptoms (total BPRS < 35). Questionnaires of Self-Rating Anxiety Scale (SAS) (Jegede, 1977) and Self-Rating Depression Scale (SDS) (Zung, 1965) were implemented to evaluate the levels of anxiety and depression for all the participants. All the participants (SAS < 60, SDS < 70) did not reach severe anxiety and depression levels. Furthermore, the International Index of Erectile Function (IIEF) (Rosen et al., 1997), which has been suggested as a primary endpoint for clinical trials of ED and as a diagnostic evaluation of ED severity (Rosen et al., 2002), was used to assess the sexual functioning for all the participants.

### Ethics statement

The study was approved by the ethics committee of Henan Provincial People's Hospital and conducted in accordance with the Helsinki Declaration. Written informed consent was obtained from all the subjects.

### MRI image acquisition

Magnetic resonance imaging (MRI) data was obtained on a 3T GE 750 Discovery MRI scanner (General Electric, Milwaukee, WI, USA), with a dedicated 8-channel head coil. High resolution structural volumes were obtained via a T1-weighted inversion recovery fast spoiled gradient recalled (IR FSPGR) sequence with the following parameters: *TR* = 8.3 ms, *TE* = 2.26 ms, flip angle = 12°, matrix resolution = 256 × 256, 160 slices, FOV = 256 × 256 mm^2^, and an isotropic resolution of 1 × 1 × 1 mm^3^.

### MR image processing and cortical thickness measurements

The MR images were processed with the CIVET MRI analysis pipeline (version 1.1.12) (Ad-Dab'bagh et al., 2006) developed at the Montreal Neurological Institute (http://www.bic.mni.mcgill.ca/ServicesSoftware/CIVET). First, native MRI images were corrected for intensity non-uniformity using the N3 algorithm (Sled et al., 1998), and images in the native space were linearly registered into the stereotaxic space (Collins et al., 1994). The registered brain volume were segmented into WM, GM, cerebrospinal fluid (CSF), and background (Zijdenbos et al., 1998). The inner and outer cortical surfaces (each hemispheric surface consisted of 40962 vertices and 81920 triangles) were then automatically extracted using the CLASP algorithm (Kim et al., 2005). The cortical surfaces were then nonlinearly aligned to a hemisphere-unbiased iterative surface template (Lyttelton et al., 2007) using a depth-potential function (Boucher et al., 2009) for accurate cross-subject correspondences. Finally, CTh was measured as the Euclidean distance between linked vertices respectively on the inner and outer cortical surfaces throughout the cortex with native scaling (Zhao et al., 2013).

### Construction of structural brain networks

**Figure 1:**
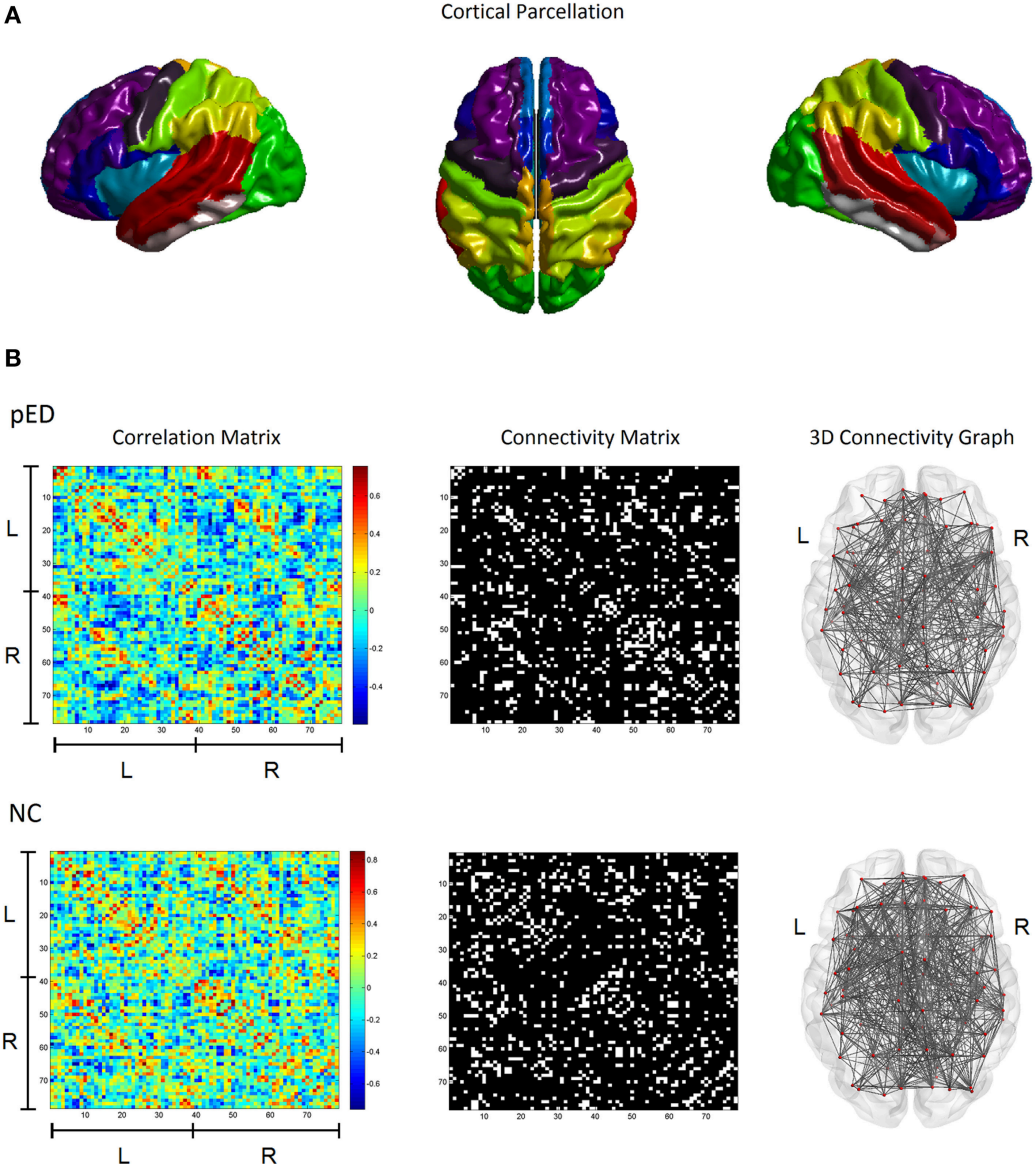
**Cortical structural covariance networks in patients with psychogenic erectile dysfunction (pED) and normal controls (NC)**. **(A)** Cortical parcellation using the AAL template. **(B)** The left column shows the cortical thickness correlation matrices of 78 AAL brain regions; the middle column shows the binary network matrices at a fixed sparsity threshold of 15%; the right column gives the 3D representations of the brain connectivity networks. L, left hemisphere; R, right hemisphere.

This work used the automatic anatomical labeling (AAL) atlas (Tzourio-Mazoyer et al., 2002) for brain parcellation (Figure 1A). Because this work is based on a cortical surface model, we included only the 78 regions of neocortex (Table S1). Subcortical structures were not included in this study. Regional CTh for each brain region was measured as the mean thickness in the region.

Structural connectivity between two brain regions was defined as their statistical similarity with respect to CTh (He et al., 2007). For each pair of brain regions, the correlation in their respective regional CTh across a group of subjects was computed using the Pearson's correlation coefficient to determine the existence of an undirected inter-regional connection. This produced a correlation matrix *C*_*ij*_ where *i*, *j* = 1, 2, …, *N*, *N* = 78 (see the left column in Figure 1B). Of note, effects of age and global mean cortical thickness (MCTh) were removed from the initial CTh measures by applying a linear regression before the correlation analysis.

For each studied group, the correlation matrix *C*_*ij*_ was thresholded into a binarized matrix *B*_*ij*_ with a given threshold in order to represent the structural brain network *G* (see the middle column in Figure 1B). For a structural brain network *G*, the nodes are the cortical regions; the undirected inter-regional connections (edges) are represented as the nonzero elements in *B*_*ij*_. To characterize the topological differences between the networks of the patients and normal controls, we used a fixed network sparsity to threshold the correlation matrix *C*_*ij*_ for both groups to ensure that the compared graphs have the same number of edges, e.g., the same wiring cost. This ensures that any remaining differences in network properties between the groups reflect differences in graph organization (Achard and Bullmore, 2007; Stam et al., 2007). The network sparsity is defined as the fraction of the number of edges in a graph compared to the maximum possible number of edges (e.g., a sparsity threshold of x% means x% of the topmost connections preserved). However, because there is currently no definitive way to select a single sparsity threshold, we therefore used a range of sparsity thresholds 5% ≤ *S*_τ_ ≤ 25%, as in earlier studies (Bassett et al., 2008; Khundrakpam et al., 2013).

### Network topological properties

Graph theory analysis (Bullmore and Sporns, 2009) was employed to compute the topological properties of the structural brain networks of pED patients and normal controls. The network topological parameters including the global efficiency *E*_global_, the local efficiency *E*_local_, the modularity *Q* and the regional efficiency *E*_regional_ were used. Each of these parameters can provide insights into the global or nodal topological properties of structural brain networks.

**Figure 2:**
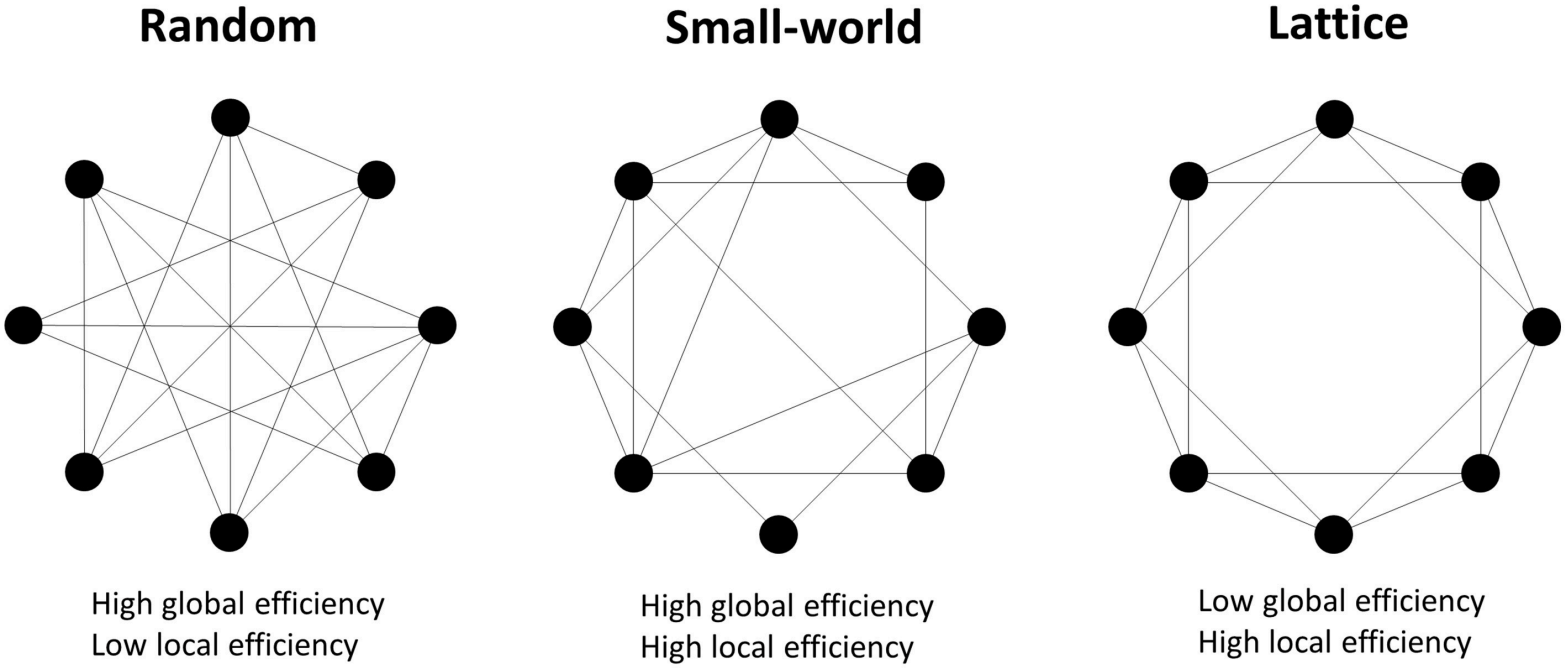
**Examples of random, small-world and lattice networks**. A small-world network possesses either long range connections between remote nodes and short range connections between neighboring nodes, which respectively contribute to high global and local network efficiencies. Small-world properties are intermediate between the random and lattice networks' properties.

The global efficiency measures the efficiency of information transfer throughout the entire network, i.e., how integrated the network is. The local efficiency measures the efficiency of segregated information transfer within subnetworks of the network, i.e., the network's ability of local specialization. For a network *G* with *N* nodes and *K* edges, the global and local efficiencies are respectively defined as Latora and Marchiori (2001):

(1) $$\begin{array}{r} E_{\text{global}}\left(G\right)=\frac{1}{N\left(N-1\right)}\sum_{i\neq j\in G}\frac{1}{d_{ij}}, \end{array}$$

(2) $$\begin{array}{r} E_{\text{local}}\left(G\right)\;=\;\frac{1}{N}\sum_{j\in G}E_{\text{global}}\left(G_{j}\right), \end{array}$$

where *d*_*ij*_ is the shortest path length between node *i* and node *j*; *G*_*j*_ is the subnetwork which contains all neighbors of node *j*. The network *G* is considered to be a small-world network if it meets the following criteria (Latora and Marchiori, 2001): *E*_global_(*G*_lattice_) < *E*_global_(*G*) < *E*_global_(*G*_random_) and *E*_local_(*G*_random_) < *E*_local_(*G*) < *E*_local_(*G*_lattice_), where *E*_global_(*G*_lattice_), *E*_global_(*G*_random_), *E*_local_(*G*_lattice_), and *E*_local_(*G*_random_) are the global and local efficiency values of lattice and random networks respectively. A random network is the network with randomly determined connections between nodes, and it has high global efficiency but low local efficiency. A lattice network refers to the network with evenly distributed connections from nodes to their neighbors, and it has low global efficiency but high local efficiency. A small-world network has higher global efficiency than a comparable lattice network and higher local efficiency than a comparable random network, i.e., is simultaneously highly integrated and segregated (Watts and Strogatz, 1998; see Figure 2).

The regional efficiency *E*_regional_ for a given node *i* is a measure of how connected the node *i* is to all other nodes in the network *G* and is defined as:

(3) $$\begin{array}{r} E_{\text{regional}}\left(i\right)\;=\;\frac{1}{N-1}\sum_{i\neq j\in G}\frac{1}{d_{ij}}. \end{array}$$

This measure quantifies the importance of a node for the communication within the network and high regional efficiency implies the hub roles (Achard et al., 2006; Achard and Bullmore, 2007). A node *i* was identified as the hub in a cortical network if *E*_regional_(*i*) was at least 1 standard deviation (SD) greater than the average *E*_regional_ of the network (i.e., *E*_regional_(*i*) > mean + SD; Gong et al., 2012).

Modularity *Q* is a measure of the degree to which a network is organized into distinct subnetworks (Newman and Girvan, 2004):

(4) $$\begin{array}{r} Q=\overset{n}{\underset{s=1}{\sum }}\left[\frac{l_{s}}{L}-{\left(\frac{d_{s}}{2L}\right)}^{2}\right], \end{array}$$

where *L* is the total number of edges of *G, n* is the number of modules in *G, l*_*s*_ is the total number of edges in module *s*, and *d*_*s*_ is the sum of the degrees (number of links connected to a node) of the nodes in module *s*.

It is noted that there is currently no definitive way to select a single sparsity threshold to construct brain structural networks. We therefore used a range of sparsity thresholds of 5% ≤ *S* ≤ 25% as in earlier studies (Bassett et al., 2008; Khundrakpam et al., 2013), and computed summarized scalars for the studied network topological properties as:

(5) $$\beta_{\text{integrated}}=\frac{\int_{a}^{b}\beta }{b-a},$$

where β is a network topological parameter, *a* and *b* are the lower and upper limits of sparsity (here, *a* = 5 and *b* = 25).

### Network analysis

The statistical significance of the between-group differences in network topological features was assessed by implementing 1000 non-parametric permutations, namely to test the null hypothesis that the observed between-group differences could occur by chance (Bullmore et al., 1999). At each permutation, group labels were randomly reassigned to all subjects, then network metrics were recalculated for the randomized groups, and differences were recomputed between the randomized groups. The critical thresholds for the one-tailed tests of the null hypothesis with the probability of type I error of 0.05 were defined with the 95 percentile points of these distributions.

## Results

### Demographic and clinical data

Demographic, psychiatric and behavioral features for the groups of pED patients and normal controls are summarized in Table 2. The two groups were not significantly different in age, education level, intracranial volume, and total BPRS score (*p* > 0.1). All the participants did not show prominent psychiatric symptoms (total BPRS < 35). The pED patients had lower IIEF score (*p* < 0.0001), and higher SAS and SDS scores (*p* < 0.005), than the normal controls. All the participants did not reach severe anxiety and depression levels (SAS < 60, SDS < 70) and did not have abnormal hormonal levels.

**Table 2:** **Demographic data and clinical variables of patients with psychogenic erectile dysfunction (pED) and normal controls (NC)**.

	**pED**	**NC**	***p*-value**
	**M ± SD**	**M ± SD**	
**DEMONGRAGHICS**			
Age (years)	28.45±6.44	30.02±3.44	0.18^a^
ICV (cm^3^)	1437.63±103.96	1451.17±91.64	0.59^a^
Education (years)	12.55±3.22	12.67±3.50	0.49^a^
**PSYCHOLOGICAL**			
Total BPRS (18-126)	20.68±0.31	20.00±0.27	0.11^a^
Total IIEF (0-75)	34.82±11.90	62.65±9.45	0.000^b^
SAS (20-80)	43.82±7.52	37.12±8.97	0.002^b^
SDS (20-80)	47.97±11.08	36.96±9.15	0.000^b^
**HORMONE**			
FSH (RR: 1.5–12.4 mIU/ml)	6.34±3.89	7.53±3.24	0.27^a^
LH (RR: 1.7–8.6 mIU/ml)	6.30±1.49	6.64±1.40	0.34^a^
PRL (RR: 4.04–15.2 ng/ml)	10.09±2.66	10.84±2.80	0.33^a^
Prog (RR: 0–1.4 ng/ml)	0.65±0.39	0.79±0.34	0.21^a^
Testo (RR: 2.8–8 ng/ml)	5.46±1.78	6.03±1.49	0.24^a^
E2 (RR: 7.63–42.6 pg/ml)	24.73±11.71	27.79±9.62	0.30^a^

*M, mean; SD, standard deviation; ICV, intracranial volume; IIEF, International Index of Erectile Function (Rosen et al., 1997); BPRS, Brief Psychiatric Rating Scale (Overall and Gorham, 1962); SAS, Self-Rating Anxiety Scale (Jegede, 1977); SDS, Self-Rating Depression Scale (Zung, 1965); RR, reference range; FSH, follicle stimulating hormone; LH, luteinizing hormone; PRL, prolactin; Prog, progesterone; Testo, Testosterone; E2, estradiol*.

a *The p-value was obtained with a two-tailed two-sample t-test*.

b *The p-value was obtained with a one-tailed two-sample t-test*.

### Inter-regional correlation in cortical thickness

Both the patient and control groups showed high correlations between contralateral homologous regions and neighboring ipsilateral regions (Table S2), which were well in line with previously reported inter-regional structural brain correlations (Mechelli et al., 2005; He et al., 2007).

### Global topology of structural cortical networks

**Figure 3:**
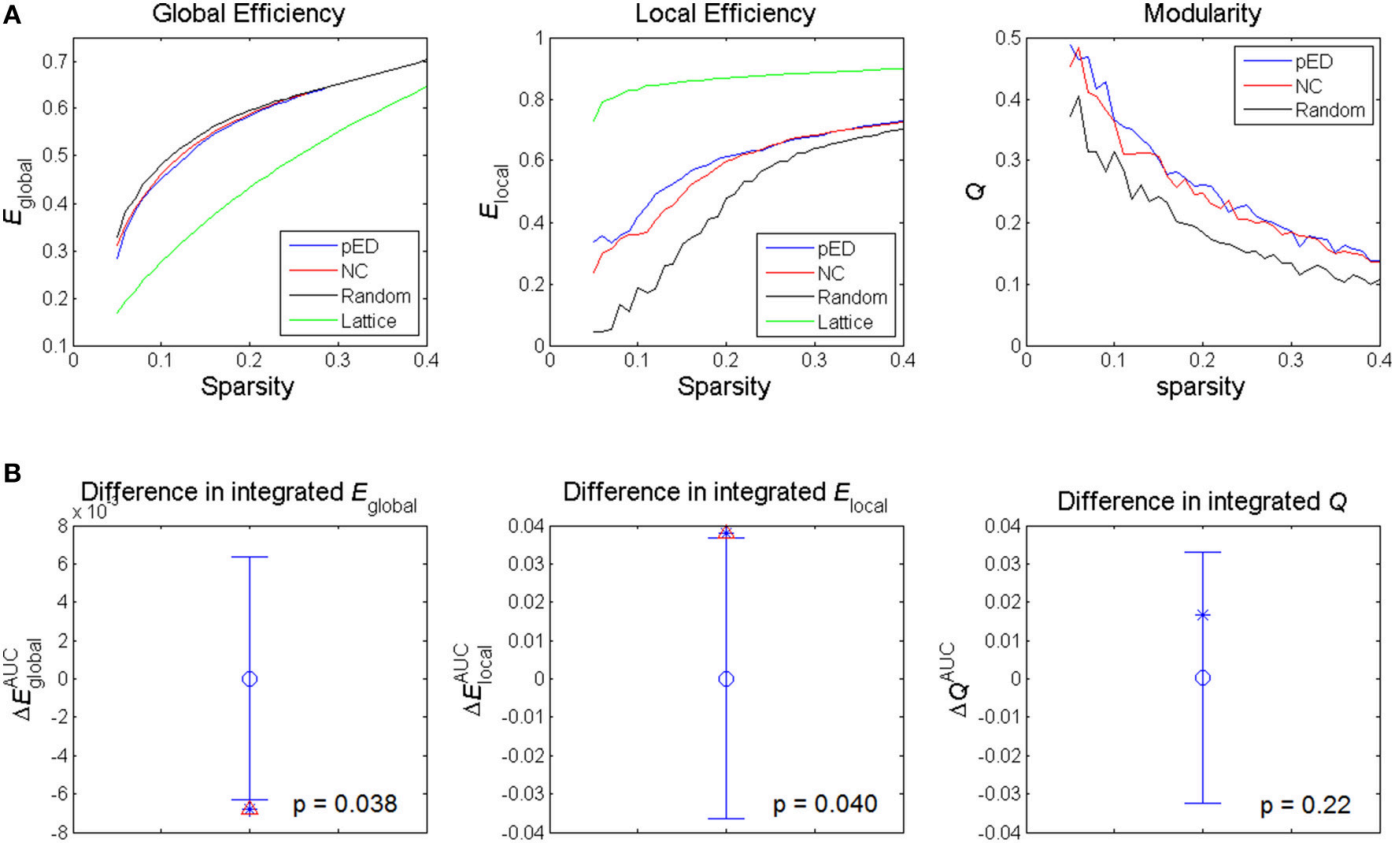
**Alterations in global network topological properties**. **(A)** Global efficiency *E*_global_, local efficiency *E*_local_, and modularity *Q* of the cortical networks of patients with psychogenic erectile dysfunction (pED) and normal control (NC) groups as a function of sparsity, and compared with simulated random and lattice networks' properties. **(B)** Differences in integrated values of the global topological parameters. The asterisks show the between-group differences (pED–NC). The mean values and 95% confidence intervals of the between-group differences acquired from 1000 permutation tests are respectively shown as open circles and lines. Significant (*p* < 0.05) differences are indicated with red triangles.

The structural brain networks of both groups of pED patients and normal controls showed a small-world organization (Watts and Strogatz, 1998), particularly in the sparsity range of 5–25%. The networks were intermediate between the simulated random network having a high global efficiency and a low local efficiency, and the simulated lattice network having a low global efficiency and a high local efficiency (Figure 3A).

The summary metrics of global network topological properties of global efficiency and local efficiency were significantly different between the two groups (Figure 3B). Compared to normal controls, pED patients showed significantly lower integrated global efficiency (*p* < 0.05, permutation test, uncorrected) and significantly higher integrated local efficiency (*p* < 0.05, permutation test, uncorrected). No significant between-group difference in the integrated modularity was found.

### Topological differences of subnetworks

**Figure 4:**
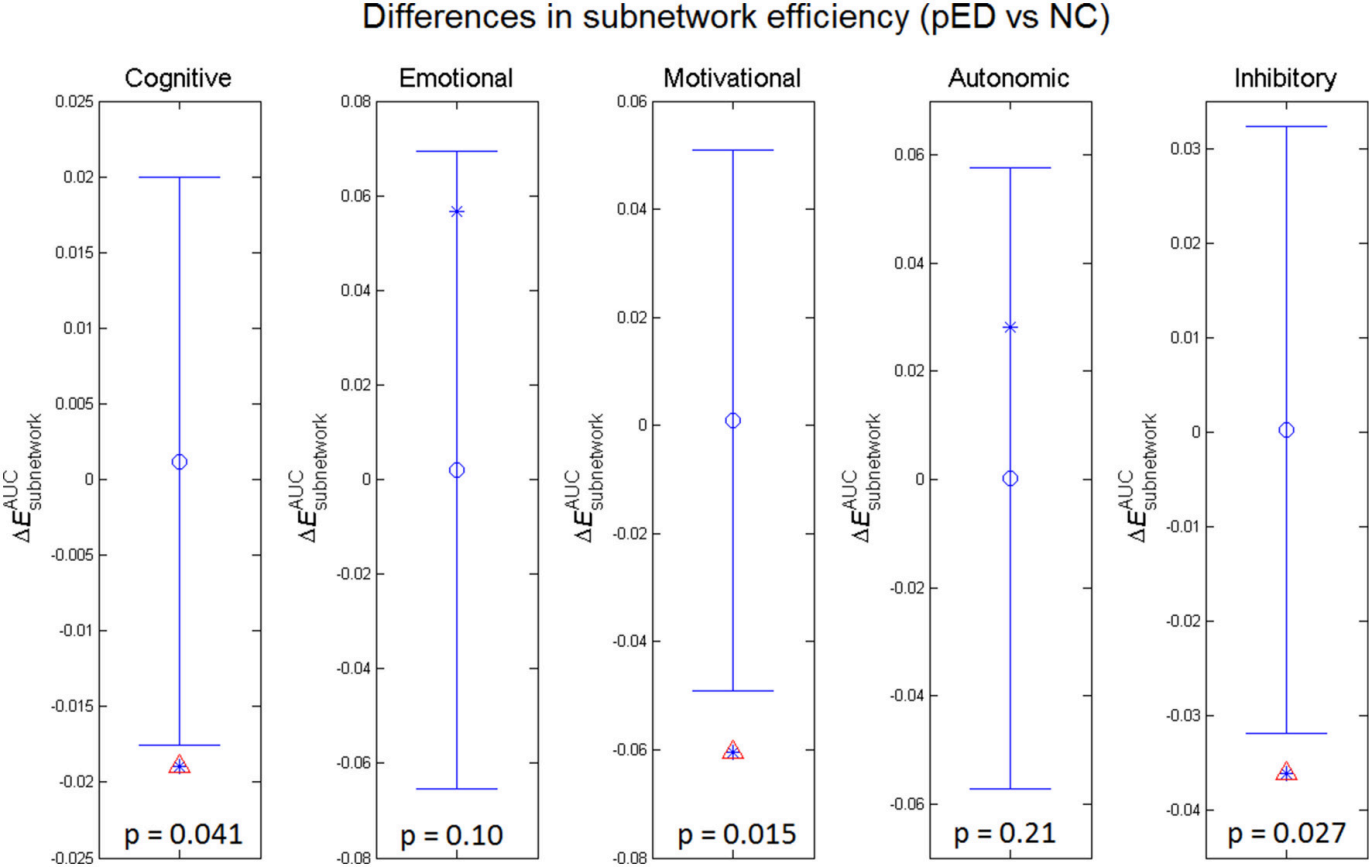
**Between-group differences in integrated subnetwork efficiency of the subnetworks for cognitive, emotional, motivational, and autonomic components involved in male sexual arousal and the subnetwork for inhibitory processes**. The asterisks show the between-group differences (pED–NC). The mean values and 95% confidence intervals of the between-group differences acquired from 1000 permutation tests are respectively shown as open circles and lines. Significant (*p* < 0.05) differences are indicated with red triangles.

To assess the relevance of the observed network topological changes with the functional dynamics of male SA, we combined the cortical regions consistently reported to be involved in the four neurobehavioral components of male SA and in the inhibitory control (Table S3), and computed the mean regional efficiency within each subnetwork (Figure 4). Compared to normal controls, the integrated mean efficiencies of the cognitive, motivational and inhibitory subnetworks for male SA were significantly decreased (*p* < 0.05, permutation test, uncorrected) in patients. These results did not survive in multiple comparison analysis using Bonferroni correction or False Discovery Rate. No significant between-group difference in the subnetwork efficiency was found for the sexual emotional and autonomic networks.

### Topological differences in nodal characteristics

**Figure 5:**
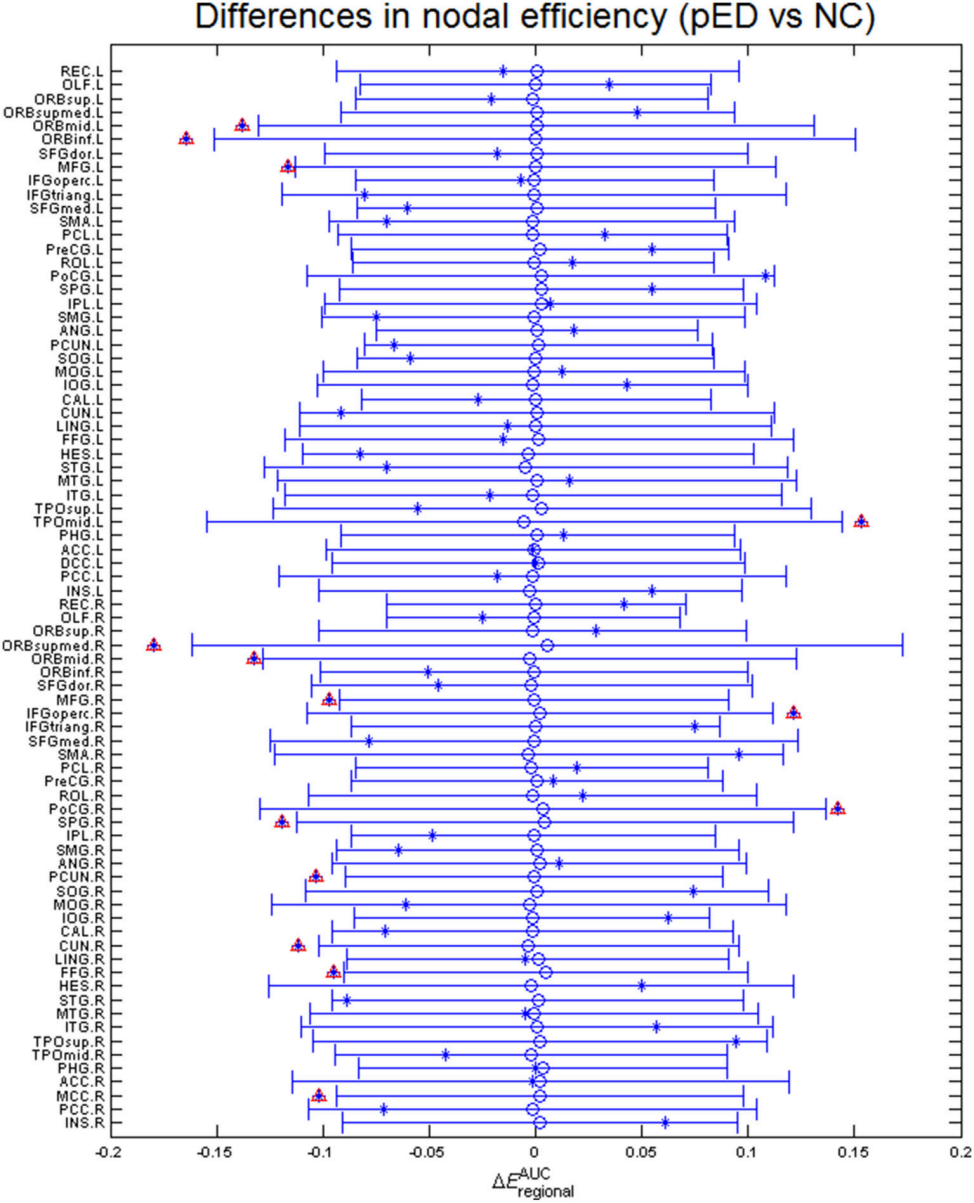
**Differences in integrated regional efficiencies of the 78 cortical regions**. The graph shows the between-group differences (pED–NC) in integrated regional efficiency as asterisks. The mean values and 95% confidence intervals of the between-group differences acquired from 1000 permutation tests are respectively shown as open circles and lines. Significant (*p* < 0.05) differences are indicated with red triangles. Decreased integrated regional efficiency was found in MFG.L, MFG.R, ORBmid.L, ORBmid.R, ORBinf.L, ORBsupmed.R, SPG.R, PCUN.R, CUN.R, FFG.R, and MCC.R; increased integrated regional efficiency was found in TPOmid.L, PoCG.R, and IFGoperc.R. L: left; R: right. For the abbreviations of regions, see Table S1.

**Figure 6:**
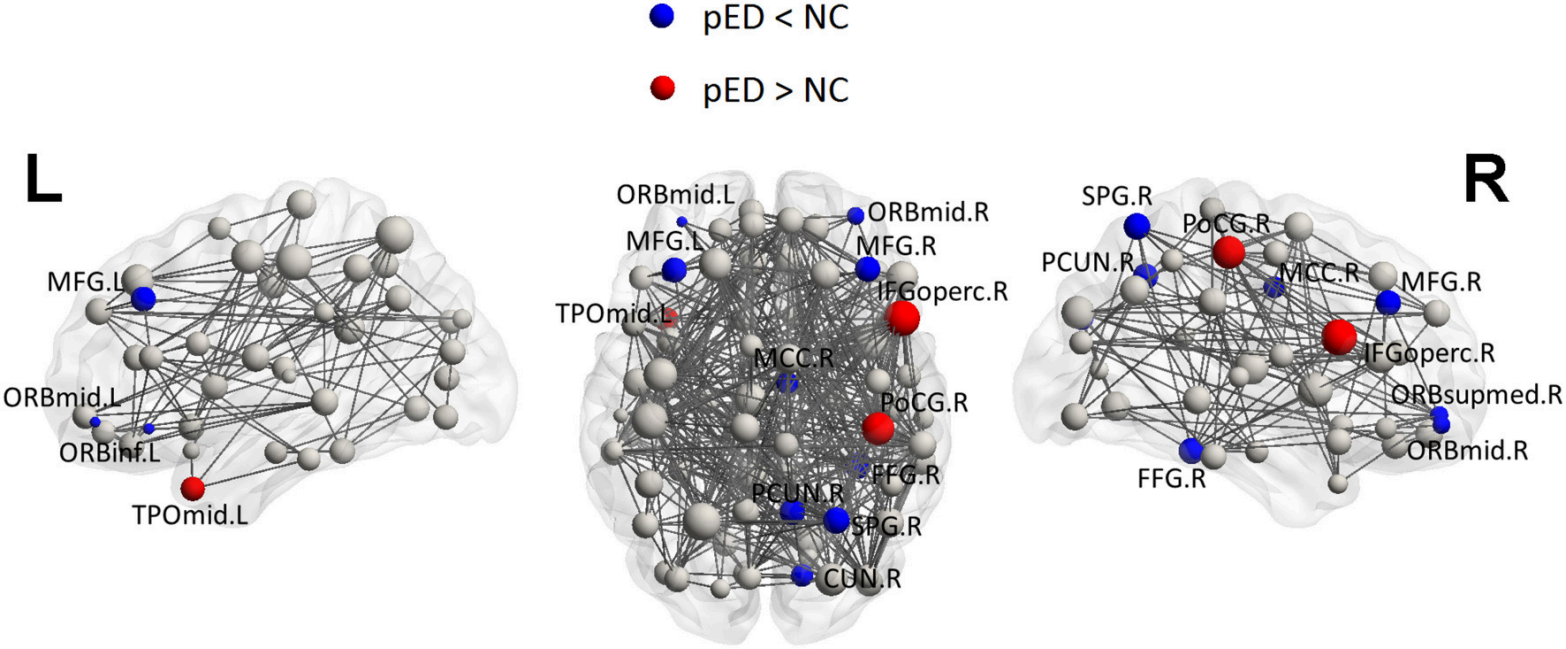
**Three Dimensional rendering maps of the integrated regional efficiencies of the group of pED patients**. Blue and red spheres respectively represent cortical regions with decreased and increased regional efficiency in pED, in comparison with normal controls. Gray spheres indicate no significant between group differences. The sizes of the spheres are proportional to the corresponding integrated regional efficiencies in pED patients. The lines represent the links of the networks with a sparsity of 15%. pED patients showed decreased integrated regional efficiency in MFG.L, MFG.R, ORBmid.L, ORBmid.R, ORBinf.L, ORBsupmed.R, SPG.R, PCUN.R, CUN.R, FFG.R, and MCC.R; and showed increased integrated regional efficiency in TPOmid.L, PoCG.R, and IFGoperc.R. L: left; R: right. For the abbreviations of regions, see Table S1.

Regional efficiencies were examined to investigate which cortical regions contributed to the abnormal global network properties in pED patients. Changes in the integrated regional efficiency for each cortical region are displayed in Figures 5, 6. pED patients showed significantly decreased regional efficiencies (*p* < 0.05, permutation test, uncorrected) in 4 regions that might mediate the cognitive component of male SA [the bilateral orbital parts of the middle frontal gyri (ORBmid), the right superior parietal gyrus (SPG) and the right fusiform gyrus (FFG)], and 1 region that might contribute to the motivational component of male SA (the right MCC), and 3 regions that might be involved in the inhibitory control of male SA [the left orbital part of the inferior frontal gyrus (ORBinf), the right medial orbital part of the superior frontal gyrus (ORBsupmed) and the right precuneus (PCUN)]. Significant reductions of regional efficiency in pED (*p* < 0.05, permutation test, uncorrected) were also found in the bilateral middle frontal gyri (MFG) and the right cuneus (CUN). Furthermore, we also observed significantly increased regional efficiencies (*p* < 0.05, permutation test, uncorrected) in the left temporal pole of the middle temporal gyrus (TPOmid), the right postcentral gyrus (PoCG) and the right opercular part of the inferior frontal gyrus (IFGoperc) in pED. None of the results remained significant after applying correction for multiple comparisons using Bonferroni correction or False Discovery Rate.

### Distribution of hubs

**Figure 7:**
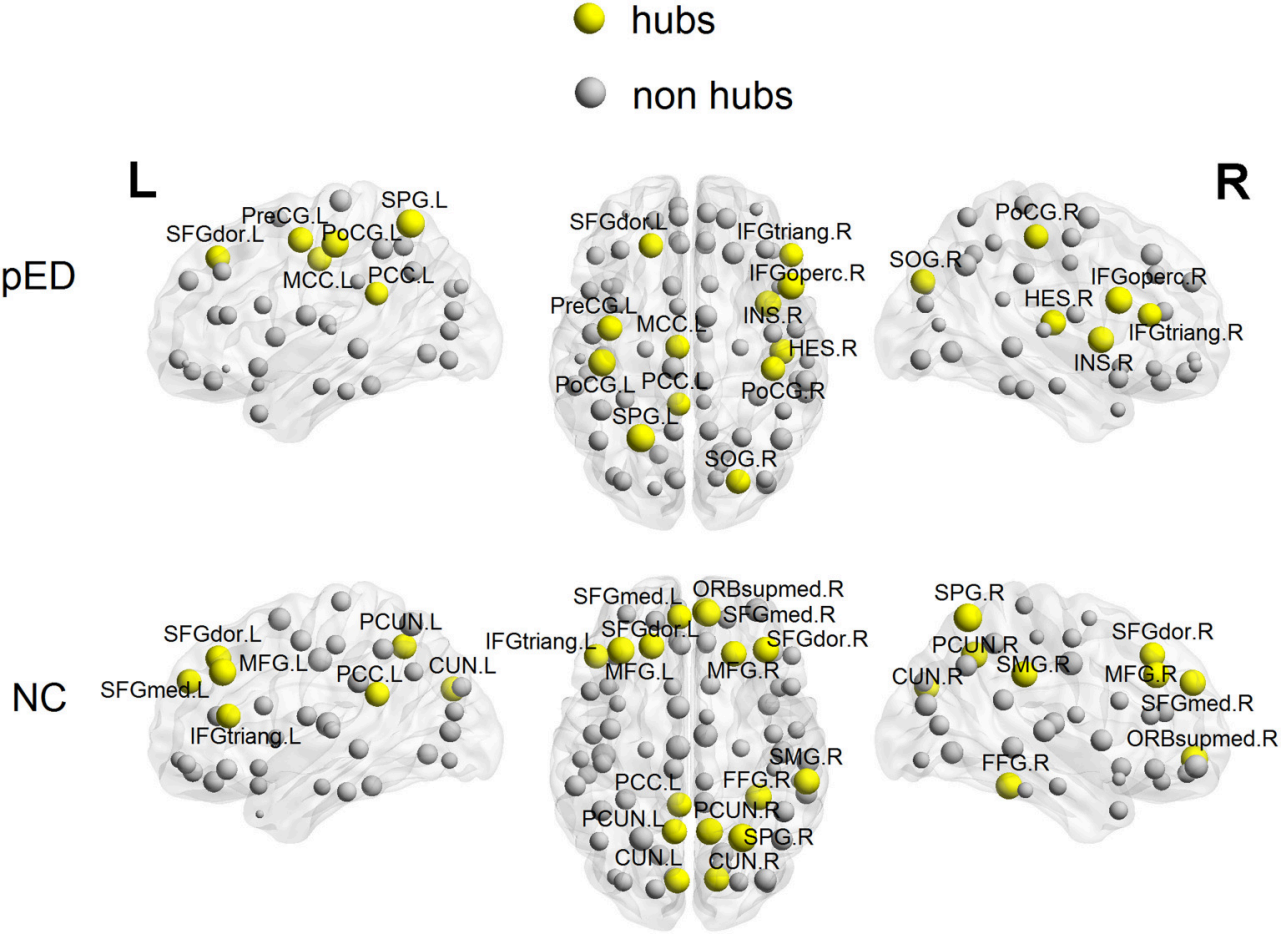
**3D rendering maps of network hubs identified in patients with psychogenic erectile dysfunction (pED) and normal controls (NC)**. Yellow and gray spheres respectively represent hub and non-hub regions. L, left; R, right. For the abbreviations of regions, see Table S1.

To put the nodal network properties of pED patients and normal controls in the global brain network topology, we show the distribution of their hub nodes in the networks (Figure 7 and Table 3). The nodes were identified as hubs if their nodal efficiencies were at least 1 SD greater than the average nodal efficiency across the network (Gong et al., 2012). Sixteen cortical regions were identified as hubs in the control group, and 12 hubs were identified in the patient group. Especially, the hubs in the control group were mainly located in the frontal and parietal cortices. This is greatly in line with previous brain network studies in healthy adults (Hagmann et al., 2008; van den Heuvel and Sporns, 2011; Gong et al., 2012). In comparison with normal controls, pED patients primarily lost hubness in the medial and lateral prefrontal (bilateral medial parts of the superior frontal gyri (SFGmed), the right ORBsupmed, the bilateral MFG), the inferior temporal (right FFG), the medial parietal (bilateral PCUN and CUN) areas; while notably showed extra hubs in the central motor (left PreCG and bilateral PoCG) and the right opercular (IFGoperc, HES and insula) areas.

**Table 3:** **Cortical regions identified as hubs in cortical networks of patients with psychogenic erectile dysfunction (pED) and normal controls (NC)**.

**pED**		**NC**	
**Regions**	**Integrated *E*_regional_**	**Regions**	**Integrated *E*_regional_**
SPG.L	0.62	SPG.R	0.63
PoCG.L	0.61	ORBsupmed.R	0.61
IFGoperc.R	0.60	MFG.L	0.61
INS.R	0.60	PCUN.R	0.60
HES.R	0.58	FFG.R	0.60
PreCG.L	0.58	SFGmed.R	0.60
SFGdor.L	0.57	MFG.R	0.60
SOG.R	0.57	SMG.R	0.60
PoCG.R	0.57	SFGdor.L	0.59
MCC.L	0.57	SFGdor.R	0.59
IFGtriang.R	0.56	CUN.R	0.58
PCC.L	0.56	PCUN.L	0.58
		CUN.L	0.58
		PCC.L	0.57
		SFGmed.L	0.57
		IFGtriang.L	0.57

*A node is identified as hub in a cortical network if E_regional_ is at least 1 standard deviation greater than the average E_regional_ of the network. L, left; R, right. For the abbreviations of regions, see Table S1*.

## Discussion

The present study, for the first time, investigated pED-related disruptions of the large-scale structural brain network topology using graph theoretical analysis. We revealed disrupted global topological organization of structural brain networks in pED patients as indicated by altered global network properties. Especially, we found that the subnetworks mediating the cognitive, motivational, and inhibitory processes of male SA were profoundly affected in pED patients; and this was supported by the aberrant nodal characteristics of certain regions belonging to these subnetworks. Overall, these findings provide evidence illustrating that the pathophysiology of pED is associated with alterations in the coordination of large-scale brain networks underlying the neurobehaviors and inhibition of male SA.

### Disrupted global topology of structural brain networks in pED

The human brain is a complex dynamical system with an optimal balance between local specialization and global integration. Small-world topology has been found in both functional and structural brain networks (Bullmore and Sporns, 2009; He and Evans, 2010), which might reflect a fundamental organizational principle in human brain networks. In consistent with these earlier studies, our study found that both pED patients and normal controls showed a small-world efficient behavior with high global and local efficiencies in structural brain networks.

Although, small-world properties were found, pED patients showed decreased global efficiency and increased local efficiency compared with normal controls. The global efficiency reflects the information transfer between remote cortical regions, and it is mainly associated with long-range connections. The local efficiency is predominantly related to short range connections between neighboring regions. Since the small-world topology of the brain reflects an optimal balance between fragmentation and coalescence (Bassett and Bullmore, 2006), our results therefore indicate a disruption of the normal balance in structural brain networks in pED. The decreased global efficiency and increased local efficiency indicate a more lattice-like organization in the brain network, which has lower ability of global integration and less efficient information propagation across the network (Strogatz, 2001). Such disrupted structural brain network topology could be due to impaired neuroanatomical (Zhang et al., 2014) and inter-regional morphometric connections (Zhao et al., 2015) or be associated with abnormal functional integrity (Cera et al., 2014) in pED.

### Disrupted subnetwork and nodal topology of brain structural networks in pED

Regional efficiency measures the extent to which a region connects all other regions of a network, and may indicate importance of the region in the whole brain network. Here, we computed the subnetwork efficiency as the mean regional efficiencies across the nodes within a specific subnetwork, and examined such metrics for the subnetworks that respectively mediate the four components of male SA and the inhibition of male SA, allowing us to assess how these subnetworks were integrated in the whole brain network. We found that subnetwork efficiencies of the cognitive, motivational and inhibitory subnetworks were significantly reduced (*p* < 0.05, permutation test, uncorrected) in pED patients compared with normal controls. These results indicate disruptive integration of these subnetworks in the brain networks, and may be related to the abnormal cognitive, motivational and inhibitory functional responses to sexually relevant stimuli in pED patients (Hagemann et al., 2003; Montorsi et al., 2003; Cera et al., 2012b).

The cognitive component of male SA comprises a process of appraisal through which stimuli are categorized or not categorized as sexual incentives and quantitatively evaluated as such. This complex analysis is conceived as mainly performed by the ITC and the OFC (Stoléru, 2014). In this work, we found that pED patients showed reduced regional efficiency (*p* < 0.05, permutation test) in the regions located at the ITC (right FFG) and the OFC (the bilateral ORBmid) compared with normal controls, which suggests the critical role of these regions in the impaired appraisal process of male SA in pED (Hagemann et al., 2003). The cognitive component of male SA also involves the activation of regions involved in sustained attention, such as the superior and inferior parietal lobules, which devotes to sexually relevant targets (Stoléru et al., 2012; Poeppl et al., 2014). Decreased regional efficiency (*p* < 0.05, permutation test) in the superior parietal lobule (the right SPG) might be related to the abnormal attentional processes in pED (Beauregard et al., 2001; Cera et al., 2012b).

The cognitive processes are interfaced by motivational processes (Stoléru, 2014), i.e., once a stimulus is perceived as sexually relevant, a motivational value gets attached to it, which makes it salient, attractive, and wanted (Robinson and Berridge, 1993). Here, we found reduced regional efficiency (*p* < 0.05, permutation test, uncorrected) in the right cingulate premotor region (the right MCC). This region has been illustrated to play a crucial role in the initiation of goal-directed behaviors (Devinsky et al., 1995), including sexual behavior (Stoléru et al., 2012). Brain activity in MCC, especially the anterior part, has been shown to be positively correlated with penile erection in healthy men; and such correlation well supported the notion that penile erection is mandatory for the initiation of copulatory behavior and thus the realization of sexual desires (Poeppl et al., 2014). Thus, our results might support the hypothesis that impairment of the sexual motivational circuits is an important factor causing psychogenic sexual dysfunction (Georgiadis and Kringelbach, 2012). However, the impairment of sexual motivational circuits in pED patients, in contrast, might also be a consequence of sexual dysfunction. It has been reported that sexual dysfunction could induce reactive negative mood effects as a consequence of unsatisfactory sexual experience (Shiri et al., 2007), which could decrease the patients' sexual desire (Lourenço et al., 2011). Specific studies on the causal relationship between the impairment of the sexual motivational network and pED are required.

Functional neuroimaging studies have revealed the existence of inhibitory mechanisms of male SA. The model holds that, in the absence of sexually relevant stimulation, brain regions, such as the medial OFC, the left lateral OFC, the LTC, the ANG, and the PCC/PCUN, may exert continuous inhibitory controls on SA; and these regions deactivate during the development of SA to release such inhibition (Stoléru et al., 2012; Poeppl et al., 2014). The nodal network characteristic analysis showed decreased regional efficiency (*p* < 0.05, permutation test, uncorrected) in the regions located at the right medial OFC (the right ORBsupmed), the left lateral OFC (the left ORBinf) and the right PCC/PCUN (the right PCUN). The medial OFC is conceived to mediate a process of devaluation of the erotic character of the sexually relevant stimuli (Stoléru et al., 2003; Stoléru, 2014). In contrast to the right lateral OFC that is thought to play a role in the cognitive component of SA, the left lateral OFC may exert a testosterone-dependent inhibitory control on SA (Redouté et al., 2005; Stoléru et al., 2012). The PCC/PCUN, a key node in the default-mode network (DMN) (Buckner et al., 2008), might be involved in the downregulation of introspective and self-reflexive processes in SA (Poeppl et al., 2014). Reduced regional efficiency in this area may be associated with its decreased functional connectivity level within the DMN in pED (Cera et al., 2014). These results support the notion that abnormalities in the inhibitory control network may play an important role in the pathology of pED (Montorsi et al., 2003; Cera et al., 2012b). It is noteworthy that, in our earlier study with the same data sample (Zhao et al., 2015), the most prominent pattern of CTh reduction in pED patients was in the right vmPFC which included the right medial OFC (ORBsupmed) identified here; and the CTh decrease in vmPFC was significantly correlated with the degrading of male sexual functioning. vmPFC has been consistently reported to play a critical role in modulating negative psychological states (Mayberg et al., 1999; Drevets et al., 2008). Such negative mood states, e.g., depression and anxiety, have been associated with male sexual dysfunction (Feldman et al., 1994; Sugimori et al., 2005). In our data sample, pED patients had lower male sexual functioning (IIEF) and higher anxiety (SAS) and depression (SDS) levels than normal controls (Table 2); and the male sexual functioning (IIEF) was negatively correlated with anxiety (SAS) and depression (SDS) levels (Pearson's *r* = −0.32, *p* < 0.01 and Pearson's *r* = −0.36, *p* < 0.005, respectively). All the evidence suggest that the reduced nodal characteristics of the medial OFC may be in favor of an adaptive cortical plasticity in vmPFC related to negative psychological effects, which might be associated with the abnormal inhibitory mechanism of male SA in pED.

The pED patients also showed decreased regional efficiency (*p* < 0.05, permutation test, uncorrected) in the nodes located at the lateral PFC (the bilateral MFG) and the visual cortex (the right CUN). It has been known that the lateral PFC is involved in multiple high-level cognitive functions, e.g., executive functions, attention and memory, whereas, its role in male SA is still not very clear. Functional connectivity studies have proposed a network comprising nodes in the lateral PFC and the parietal cortex for attentional processes (Corbetta and Shulman, 2002). The altered nodal characteristics in the lateral PFC, together with the reduced regional efficiency in the superior parietal lobule discussed above, may indicate the disruption of the frontoparietal attentional network in pED patients. There is also some evidence that the lateral PFC is implicated in inhibition of male SA (Beauregard et al., 2001); relating to this, feelings of guilt and shame might be represented in this area as well (Michl et al., 2014). Thus, it is also plausible that the nodal topological changes observed in the lateral PFC might be related to the abnormal inhibitory control of male SA in pED. Activations of the visual cortex responding to visual sexual stimuli have been consistently found in healthy men (Beauregard et al., 2001; Arnow et al., 2002; Mouras et al., 2003), which, however, may not be specifically related to sexual condition (Mouras et al., 2003). Moreover, existing functional neuroimaging studies on male sexual dysfunction did not observe abnormal functional activities in the visual cortex in patients. Thus, it is not clear whether the altered nodal characteristic in the right visual cortex is associated with pED, and it needs caution to interpret this result.

Taken together, the altered nodal properties may cause a segregation of the systems mediating the cognitive and motivational components of male SA and the inhibitory control of male SA, and yield a disruptive integration of the brain networks. This is consistent with the altered subnetwork properties discussed before.

In this work, we also observed increased regional efficiencies (*p* < 0.05, permutation test, uncorrected) in the left temporal pole (TPOmid), the right primary somatosensory cortex (PoCG) and the right vPM (IFGoperc) in pED patients. The temporal pole has been proposed to bind complex, highly processed perceptual inputs to visceral emotional responses (Olson et al., 2007). The primary somatosensory cortex, whose upper part receives inputs form the external genitalia and lower part may be involved in somatosensory imagery and representation (Hu et al., 2008; Keysers et al., 2010), is a key node in network mediating the emotional component of male SA (Stoléru et al., 2012). The vPM is an important hub in both the sexual motor imagery network (Mouras et al., 2003; Stoléru et al., 2003) and the mirror-neuron system (Mouras et al., 2008). In healthy men, penile erection has been significantly associated with activity in this area (Poeppl et al., 2014). However, previous studies did not observe abnormal dynamics in these regions in pED patients (Montorsi et al., 2003; Cera et al., 2012b; Liu et al., 2015), thus they might retain their functional abilities in pED. In addition, our earlier study with the same data sample in Zhao et al. (2015) also provided the evidence of increased connectivity from the right vPM with the right insula in pED patients. Thus, it could be speculated that the increased nodal efficiency in these regions might indicate a complementary mechanism for the observed reduction of nodal efficiency in other regions described before. However, complex studies utilizing multi-modal neuroimaging data are needed to verify such inference.

In addition, high nodal efficiency implies a hub role for the node (Achard et al., 2006; Achard and Bullmore, 2007). In our data, normal controls showed a distribution of hubs primarily in the prefrontal and parietal cortices, which is largely consistent with previous brain network studies (Hagmann et al., 2008; van den Heuvel and Sporns, 2011; Gong et al., 2012). However, compared with controls, pED patients showed loss of medial and lateral prefrontal (SFGmed, ORBsupmed, MFG), inferior temporal (FFG) and medial parietal hubs (PCUN), which are key nodes in the inhibitory, cognitive and motivational networks for male SA. Extra central motor (e.g., bilateral PoCG), and right opercular (e.g., IFGoperc and insula) hubs were observed in patients. These regions that showed abnormal hubness also showed abnormal CTh in pED in our earlier study (Zhao et al., 2015), and are well in line with the between-group differences in nodal efficiency described above. It has been suggested that brain hubs should have longer-range and more metabolically costly functional connections, so that they are likely to be more susceptible to metabolic insult and might provide a conceptually potential pathogenetic mechanism of dysfunction (Wang et al., 2010). Therefore, the hub topology in patients observed here further supports the association between the pathology of pED and the alterations in patterns of efficiency in brain networks.

Of note, current findings about the altered topological patterns of structural brain networks in pED are well in line with our previous study with the same data sample in Zhao et al. (2015), where many regions that showed reduced nodal characteristics in pED here, such as vmPFC, OFC, MCC, ITC, and PCC/PCUN regions, were found to show reduced CTh, and the CTh reduction was significantly correlated with male sexual functioning degradation. Moreover, in the earlier study, we found that pED patients had decreased inter-regional CTh correlations from the right lateral OFC to the right SMG and the left ANG, which may imply disassociations from the cognitive network of male SA with the motivational and inhibitory networks of male SA in pED. Therefore, the altered topological patterns of structural brain networks in pED observed in this work are supported by the alterations in inter-regional CTh correlations.

### Methodological limitations and future work

Several issues in the present work need to be addressed. First, the structural brain networks were constructed based on inter-regional CTh correlations across subjects in groups of pED patients and normal controls. Therefore, the correlations of network topological properties and clinical factors, e.g., disease duration and erectile performance, and psychiatric scores, e.g., depression and anxiety, could not be investigated at an individual level. It is desirable to obtain individual-level measurements of network properties using, e.g., resting-state functional MRI (Achard et al., 2006; Liu et al., 2008) and diffusion-weighted imaging (Gong et al., 2009; Reijmer et al., 2013) data. Especially, combining multi-modal neuroimaging approaches would facilitate a more comprehensive understanding of the association between abnormalities in the brain network organization and functional impairments. Second, the significance of the detected between-group differences in network properties was marginal and was presented with a critical threshold of *p* < 0.05 at uncorrected level. None of the results survived correction for multiple comparisons using Bonferroni correction or False Discovery Rate. This might be because that the employed permutation test for between-group comparisons was conservative and strict, and the effect size of abnormalities in network topological properties in our sample was not large enough to survive correction for multiple comparisons. However, our results, for the first time, illustrated that changes in brain network topology might be associated with pED, and were well in line with existing findings of deficits in brain connectomes (Cera et al., 2014; Zhang et al., 2014; Zhao et al., 2015) and disruptions in neurobehavioral networks of male SA (Cera et al., 2012b) in pED. Third, this work used the anatomical AAL atlas for cortical parcellation, in which a priori anatomical classifications were defined. Since the relationship between anatomical and functional anatomies is not precisely known, the regional boundaries in the atlas may not be able to provide accurate functional segmentations so that some functional subdivisions might not be included or separated. Using an alternative atlas that has higher resolution and accurate functional segmentations is desirable in the future. Moreover, subcortical regions were not included as this study was based on a cortical surface model. It has been revealed that subcortical structures also play important role in the central mechanisms controlling male SA. Thus, it will be a meaningful future work to extend the study to subcortical regions to explore their contributions to the disruption of the brain network organization in pED.

## Conclusions

In conclusion, we, for the first time, investigated the topological organization of large scale structural brain networks in pED patients using graph theory analysis. Compared with healthy men, pED patients showed a disturbance of the optimal topological organization reflected by a shift toward a more lattice-like organization, i.e., lower global efficiency and higher local efficiency. This indicates that the brain networks in pED patients may have disrupted neural integrations among distant regions that may be associated with impaired dynamic controls of male SA. Such result is supported by the impaired neuroanatomical and morphometric connections (Zhang et al., 2014; Zhao et al., 2015) and abnormal functional integrity (Cera et al., 2014) previously reported in pED. Moreover, our subnetwork and nodal analyses revealed that the disrupted global brain network topology in pED was primarily relevant to reduced subnetwork and nodal efficiencies within the networks mediating the cognitive, motivational and inhibitory components of male SA, which indicate disruptive integration of these networks in the whole brain networks. In addition, the abnormal hubness observed in the regions involved in the cognitive, motivational and inhibitory processes of male SA further revealed the important roles they may play in the pathology of pED. Moreover, it has been conceived that these neurobehavioral components of male SA are closely interrelated and coordinated, and the cognitive component is the first step in the whole development of male SA with later processes depending on it (Stoléru et al., 2012). Thus, our results might account for the decreased cognitive and motivational activations and the extended inhibitory control of male SA previously observed in pED patients during sexually relevant stimuli (Hagemann et al., 2003; Montorsi et al., 2003; Cera et al., 2012b). Overall, the current work provides evidence for disrupted integrity in large-scale brain networks underlying the neurobehavioral processes of male SA in pED and provides new insights into the understanding of the pathophysiological mechanisms of pED at a systematic level.

## Funding

This work was supported by the Canadian Institutes of Health Research (CIHR-MOP 37754) and Chinese Nature Science Foundation (Grant No. 81271534). The funding organizations had no influence on the design and conduct of the study; collection, management, analysis, and interpretation of the data; and preparation, review, or approval of the manuscript.

### Conflict of interest statement

The authors declare that the research was conducted in the absence of any commercial or financial relationships that could be construed as a potential conflict of interest.
