# Supplementary material for: Aberrant Topological Patterns of Structural Cortical Networks in Psychogenic Erectile Dysfunction
Source: Front Hum Neurosci. 2015 Dec 18;9:675. doi: 10.3389/fnhum.2015.00675 (PMC4683194; doi:10.3389/fnhum.2015.00675)
Supplement: Supplementary file 1 [file Table1.DOCX]

**Table S1:** Cortical surface regions defined by automatic anatomical labeling (AAL) template in standard stereotaxic space.

| **Brain Regions** | **Description** |
| --- | --- |
| REC.L  REC.R | Left Gyrus Rectus  Right Gyrus Rectus |
| OLF.L  OLF.R | Left Olfactory Cortex  Right Olfactory Cortex |
| ORBsup.L  ORBsup.R | Left Superior Frontal Gyrus, Orbital part  Right Superior Frontal Gyrus, Orbital part |
| ORBsupmed.L  ORBsupmed.R | Left Superior Frontal Gyrus, Medial Orbital  Right Superior Frontal Gyrus, Medial Orbital |
| ORBmid.L  ORBmid.R | Left Middle Frontal Gyrus, Orbital part  Right Middle Frontal Gyrus, Orbital part |
| ORBinf.L  ORBinf.R | Left Inferior Frontal Gyrus, Orbital part  Right Inferior Frontal Gyrus, Orbital part |
| SFGdor.L  SFGdor.R | Left Superior Frontal Gyrus, Dorsolateral  Left Superior Frontal Gyrus, Dorsolateral |
| MFG.L  MFG.R | Left Middle Frontal Gyrus  Right Middle Frontal Gyrus |
| IFGoperc.L  IFGoperc.R | Left inferior Frontal Gyrus, Opercular part  Right inferior Frontal Gyrus, Opercular part |
| IFGtriang.L  IFGtriang.R | Left inferior Frontal Gyrus, Triangular part  Right inferior Frontal Gyrus, Triangular part |
| SFGmed.L  SFGmed.R | Left Superior Frontal Gyrus, Medial  Right Superior Frontal Gyrus, Medial |
| SMA.L  SMA.R | Left Supplementary Motor Area  Right Supplementary Motor Area |
| PCL.L  PCL.R | Left Paracentral Lobule  Right Paracentral Lobule |
| PreCG.L  PreCG.R | Left Precentral Gyrus  Right Precentral Gyrus |
| ROL.L  ROL.R | Left Rolandic Operculum  Right Rolandic Operculum |
| PoCG.L  PoCG.R | Left Postcentral Gyrus  Right Postcentral Gyrus |
| SPG.L  SPG.R | Left Superior Parietal Gyrus  Right Superior Parietal Gyrus |
| IPL.L  IPL.R | Left Inferior Parietal, but Supramarginal and Angular Gyri  Right Inferior Parietal, but Supramarginal and Angular Gyri |
| SMG.L  SMG.R | Left Supramarginal Gyrus  Right Suprmarginal Gyrus |
| ANG.L  ANG.R | Left Angular Gyrus  Right Angular Gyrus |
| PCUN.L  PCUN.R | Left Precuneus  Right Precuneus |
| SOG.L  SOG.R | Left Superior Occipital Gyrus  Right Superior Occipital Gyrus |
| MOG.L  MOG.R | Left Middle Occipital Gyrus  Right Middle Occipital Gyrus |
| IOG.L  IOG.R | Left Inferior Occipital Gyrus  Right Inferior Occipital Gyrus |
| CAL.L  CAL.R | Left Calcarine fissure and surrounding cortex  Left Calcarine fissure and surrounding cortex |
| CUN.L  CUN.R | Left Cuneus  Right Cuneus |
| LING.L  LING.R | Left Lingual Gyrus  Right Lingual Gyrus |
| FFG.L  FFG.R | Left Fusiform Gyrus  Right Fusiform Gyrus |
| HES.L  HES.R | Left Heschl Gyrus  Right Heschl Gyrus |
| STG.L  STG.R | Left Superior Temporal Gyrus  Right Superior Temporal Gyrus |
| MTG.L  MTG.R | Left Middle Temporal Gyrus  Right Middle Temporal Gyrus |
| ITG.L  ITG.R | Left Inferior Temporal Gyrus  Right Inferior Temporal Gyrus |
| TPOsup.L  TPOsup.R | Left Temporal Pole: Superior Temporal Gyrus  Right Temporal Pole: Superior Temporal Gyrus |
| TPOmid.L  TPOmid.R | Left Temporal Pole: MiddleTemporal Gyrus  Right Temporal Pole: Middle Temporal Gyrus |
| PHG.L  PHG.R | Left Parahippocampal Gyrus  Right Parahippocampal Gyrus |
| ACC.L  ACC.R | Left Anterior Cingulate and Paracingulate Gyri  Right Anterior Cingulate and Paracingulate Gyri |
| MCC.L  MCC.R | Left Median Cingulate and Paracingulate Gyri  Right Median Cingulate and Paracingulate Gyri |
| PCC.L  PCC.R | Left Posterior Cingulate Gyrus  Right Posterior Cingulate Gyrus |
| INS.L  INS.R | Left Insula  Right Insula |
