# Supplementary material for: Aberrant Topological Patterns of Structural Cortical Networks in Psychogenic Erectile Dysfunction
Source: Front Hum Neurosci. 2015 Dec 18;9:675. doi: 10.3389/fnhum.2015.00675 (PMC4683194; doi:10.3389/fnhum.2015.00675)
Supplement: Supplementary file 2 [file Table2.DOCX]

**Table S2A:** Top 10 significant correlations in patients with psychogenic erectile dysfunction.

| Region(i) | Region(j) | Correlation  coefficient (r) | p-value |
| --- | --- | --- | --- |
| REC.L | ORBsup.L | 0.76 | 7.95e-9 |
| REC.L | ORBsupmed.L | 0.75 | 1.23e-8 |
| SFGdor.L | MFG.L | 0.71 | 1.37e-7 |
| PreCG.L | PoCG.L | 0.68 | 5.61e-7 |
| SPG.L | SPG.R | 0.68 | 8.16e-7 |
| SPG.R | IPL.R | 0.67 | 1.02e-6 |
| IPL.R | ANG.R | 0.66 | 1.63e-6 |
| STG.R | MTG.R | 0.66 | 1.80e-6 |
| TPOsup.R | TPOmid.R | 0.65 | 3.26e-6 |
| IFGoperc.R | INS.R | 0.64 | 3.73e-6 |

**Table S2B:** Top 10 significant correlations in normal controls.

| Region(i) | Region(j) | Correlation  coefficient (r) | p-value |
| --- | --- | --- | --- |
| SFGdor.L | MFG.L | 0.86 | 1.67e-12 |
| SFGdor.L | SFGmed.L | 0.83 | 2.81e-11 |
| ORBsup.R | ORBinf.R | 0.83 | 2.91e-11 |
| SFGdor.R | MFG.R | 0.82 | 6.60e-11 |
| SFGdor.R | SFGmed.R | 0.82 | 7.10e-11 |
| MFG.R | SFGmed.R | 0.77 | 6.07e-9 |
| SPG.L | SPG.R | 0.76 | 1.02e-8 |
| PCUN.L | PCUN.R | 0.76 | 1.36e-8 |
| CUN.L | CUN.R | 0.75 | 1.55e-8 |
| MCC.L | MCC.R | 0.74 | 4.16e-8 |
