# Supplementary material for: Aberrant Topological Patterns of Structural Cortical Networks in Psychogenic Erectile Dysfunction
Source: Front Hum Neurosci. 2015 Dec 18;9:675. doi: 10.3389/fnhum.2015.00675 (PMC4683194; doi:10.3389/fnhum.2015.00675)
Supplement: Supplementary file 3 [file Table3.DOCX]

**Table S3:** Cortical regions (AAL parcellation) related to the cognitive, emotional, motivational and autonomic components and the inhibitory control of normal male sexual arousal, which were summarized based on the meta-analyses in ([Stoleru et al., 2012](#_ENREF_52), [Poeppl et al., 2014](#_ENREF_41)).

| **Cognitive** | **Emotional** | **Motivational** | **Autonomic** | **Inhibitory** |
| --- | --- | --- | --- | --- |
| IFGoperc.L, IFGtriang.L,  SMA.L, SPG.L,  IPL.L,FFG.L, ORBinf.R, IFGoperc.R,  IFGtriang.R, SPG.R, IPL.R, FFG.R | PoCG,L, INS.L, PoCG.R, INS.R | SMG.L, MCC.L, SMG.R, MCC.R | ACC.L, INS.L, ACC.R, INS.R | REC.L, ORBsupmed.L, ORBinf.L, ANG.L, PCC.L, PCUN.L, STG.L, MTG.L, ITG.L, REC.R, ORBsupmed.R, ANG.R, PCC.R, PCUN.R, STG.R, MTG.R, ITG.R |

For the abbreviations of regions, see supplemental Table S1.
